# Harnessing the Electrochemical Effects of Electroporation-Based Therapies to Enhance Anti-tumor Immune Responses

**DOI:** 10.1007/s10439-023-03403-x

**Published:** 2023-11-21

**Authors:** Zaid S. Salameh, Kenneth N. Aycock, Nastaran Alinezhadbalalami, Khan Mohammad Imran, Iain H. McKillop, Irving C. Allen, Rafael V. Davalos

**Affiliations:** 1 Department of Biomedical Engineering and Mechanics, Virginia Tech, 325 Stanger St, Blacksburg, VA 24061 USA; 2 Department of Biomedical Sciences and Pathobiology, VA-MD College of Veterinary Medicine, Virginia Tech, 205 Duck Pond Drive, Blacksburg, VA 24061 USA; 3 Department of Surgery, Atrium Health Wake Forest Baptist Medical Center, 1000 Blythe Blvd, Charlotte, NC 28203 USA; 4 Wallace H. Coulter Department of Biomedical Engineering, Georgia Tech - Emory, 313 Ferst Dr, Atlanta, GA 30308 USA

**Keywords:** Irreversible electroporation, Electroporation, Immunology, Electrochemical, Anti-tumor, pH, Direct current, Warburg effect, Tumor-associated macrophages, Hepatocellular carcinoma, Ablation, THP-1, Immunosuppressive, Tumor microenvironment

## Abstract

This study introduces a new method of targeting acidosis (low pH) within the tumor microenvironment (TME) through the use of cathodic electrochemical reactions (CER). Low pH is oncogenic by supporting immunosuppression. Electrochemical reactions create local pH effects when a current passes through an electrolytic substrate such as biological tissue. Electrolysis has been used with electroporation (destabilization of the lipid bilayer via an applied electric potential) to increase cell death areas. However, the regulated increase of pH through only the cathode electrode has been ignored as a possible method to alleviate TME acidosis, which could provide substantial immunotherapeutic benefits. Here, we show through *ex vivo* modeling that CERs can intentionally elevate pH to an anti-tumor level and that increased alkalinity promotes activation of naïve macrophages. This study shows the potential of CERs to improve acidity within the TME and that it has the potential to be paired with existing electric field-based cancer therapies or as a stand-alone therapy.

## Introduction

Acidity (low pH) is an oncogenic characteristic of the tumor microenvironment, supporting immunosuppression and tumor expansion.^13^ Low pH arises from increased production of lactate and hydrogen ions in malignant cells that increasingly rely on aerobic glycolysis (Warburg effect).^18^ At a lower pH, T lymphocytes and natural killer (NK) cell function decreases and cells may become apoptotic.^4,20^ Conversely, immunosuppressive cells (regulatory T cells) activate^42^ and tumor-associated macrophages (TAMs) transform into a pro-tumor phenotype.^11^ Therefore, tumor acidity is a critical regulator of cancer immunity that orchestrates both local and systemic immunosuppression,^8^ providing a need for therapeutic targets.

Previous studies have targeted tumor pH using oral buffers (sodium bicarbonate) to elevate the TME pH and encourage immune cell infiltration.^33^ While effective in preventing metastases, sodium bicarbonate therapy does not address the primary tumor when used as a monotherapy.^36^ Recently, a combinatorial therapy of ethanol ablation (to treat the primary tumor) in conjunction with oral sodium bicarbonate (to elevate tumor pH) and cyclophosphamide (to deplete regulatory T cells) proved effective in treating the primary tumor and in preventing metastases.^25^

Hepatocellular Carcinoma (HCC), one of the many neoplasms associated with chronic inflammation (and an oncogenic TME),^7^ leads to sustained changes in both the innate hepatic immune response and systemic immune cell infiltration.^34^ Surgical resection (transplant or partial hepatectomy) currently provides the best clinical strategy to treat HCC patients but can be limited by late diagnosis, tumor size and/or location, underlying pathology, and lack of organs for transplant.^2^ Although thermal ablation (radiofrequency and microwave ablation (RFA/MWA)) has emerged as a viable alternative to resection for liver neoplasms,^19^ the indiscriminate tissue damage arising within the ablative zone can lead to challenges when ablating tumors located near critical structures.

Irreversible electroporation (IRE) has emerged as an alternative to thermal ablation.^9^ With IRE systems a high voltage electrical potential is delivered in short pulses (80-100 μs) across the target region between appropriately placed electrodes leading to the formation of nanodefects in the lipid bilayer of cells within the electric field. These nanodefects can lead to loss of homeostasis and induce cell death pathways.^1^ Unlike thermal ablation, IRE selectively induces cell death within the ablation zone without damage to the underlying tissue architecture, preventing damage to structures such as blood vessels, ducts, and nerves.^22,32^ Notably, due to its non-thermal cell death mechanism, IRE treatment initiates a robust anti-tumor response by preserving antigen presentation.^3,14,35^

Electrochemical reactions that occur proximal to the electrodes in IRE treatments alter the tissue’s pH immediately around the cathode (alkaline) and anode (acidic).^41^ Others have exploited electrolysis to generate toxic byproducts to increase ablation volumes by taking advantage of the reversible electroporation regime.^16,31^ In such cases, the electrochemical reactions are maximized through monophasic, long (or DC), low voltage pulses, or high-charge exponentially decaying pulses.^37^ Importantly, electrolytic electroporation uses bipolar geometries in which both the cathode and anode contribute to the formation of electrolytic byproducts. We hypothesize that through proper pulse parameter selection and electrode geometry we could exploit these electrochemical reactions to elevate the pH and reduce the immunosuppressive nature of the TME.

Here, we propose a combinatorial therapy to target the primary tumor (IRE) and then elevate the local pH through electrochemical reactions to support the anti-tumor immune response. We first study the impact of DC pulse parameters and electrode geometries on tissue pH, then examine the impact of pH changes on THP-1 macrophage activation, before demonstrating in a proof-of-concept experiment that the pulsing strategy developed herein can re-establish a homeostatic electrochemical TME. Agar tissue mimics visualized the electrochemical effects via a pH indicator dye, flow cytometry analysis of THP-1 cells revealed the effect of elevated pH on a representative immune cell (macrophages), and *ex vivo* porcine liver provided a clinically relevant domain to test our combinatorial therapy because it is analogous to HCC, a plausible treatment target. Our results demonstrate that long, low voltage pulses applied through the cathode terminal successfully increases the pH of liver tissue to an anti-tumor level (as determined by in vitro studies), validating the potential use of electrolysis to treat acidosis within the TME and promote an anti-tumor immune response.

## Materials and Methods

### Area of pH Change

#### Agarose Tissue Phantom Preparation

A 1% (*w/v*) agar (ThermoFisher, Waltham, MA) solution was prepared in deionized water (90 °C) and 10% (*v/v*) bromothymol blue (pH indicator) added (ThermoFisher). To control electrical conductivity, NaCl was added in 0.1% (*w/v*) increments (final NaCl concentration; 0.1–1.0%) and phantoms were allowed to solidify overnight (room temperature).

#### Pulse Parameters for Increasing Voltage or Current Delivery

Pulse width (10 ms), pulse number (100), and frequency (1 Hz) were held constant while voltage or current was increased (*i.e.,* current was maintained with increasing voltage or *vice versa*) in the NaCl containing phantoms. A single pre-pulse (10 V, 50 µs length) was delivered to each phantom to determine initial resistance and Ohm’s law used to determine the change in voltage (or current) required to deliver constant current (or maintain a predetermined voltage).

#### Increasing Pulse Width

Current (100 mA), voltage (100 V), and frequency (1 Hz) were held constant while pulse width was increased (100 µs − 1 s). In doing so, the generator was unable to store sufficient charge to deliver a 1 s pulse at 100 V, so the voltage was dropped to 10 V for the 1 s pulse width and the frequency decreased to 0.5 Hz (allowing a 1 s interval between pulses).

#### Pulse Delivery and pH Measurement

Treatments were applied to phantoms using an ECM 830 Square Wave Electroporator (Harvard Apparatus, Holliston, MA). Current and voltage readings were collected using a wideband current monitor (Pearson Electronics, Palo Alto, CA) connected to an oscilloscope (Wavesurfer 3024z, Teledyne LeCroy, Chestnut Ridge, NY). Each treatment was applied using a custom needle-grounding ring electrode configuration. Representative images were captured and area of alkaline pH (pH > 7.6 = blue) was quantified using ImageJ software (NIH, Bethesda, MD).

#### Effect of Probe Number and Geometry

Number of electrodes (2-4) and arrangement was varied; When two electrodes were used the anode and cathode remained constant. When three electrodes were used, each of the three possible electrode pairs was used for one third of the treatment protocol. When four electrodes were used, each of the 6 possible electrode pairs was used for one sixth of the treatment. For groups in which a grounding ring was included, all the electrodes were connected to the cathode and the grounding ring connected to the anode. Energy matched IRE (1000 V at 100 μs) and longer, lower voltage pulse settings (10 V at 1 s) per pulse were compared.

#### Cell Culture

Purified THP-1 monocytes (TIB-202, ATCC, Manassas, VA) were cultured (2 × 10^5^–8 × 10^5^ cells/mL) in RPMI-1640 medium supplemented with 10% (*v/v*) FBS, 1% (*v/v*) penicillin/streptomycin, and 0.05 mM 2-mercaptoethanol. Differentiation of THP-1 monocytes was induced by the addition of 150 nM phorbol 12-myristate 13-acetate (PMA) (24 hours) followed by replacement with PMA-free culture medium for a further 24 hours. To induce M1 activation, differentiated THP-1 macrophages were cultured in the presence of IFN-γ (20 ng/mL) and lipopolysaccharide (LPS; 1 ng/mL) for 48 hours. To induce M2 activation, differentiated THP-1 macrophages were cultured in the presence of interleukin-4 (IL-4; 20 ng/mL) and interleukin-13 (IL-13; 20 ng/mL) for 48 hours.

#### Effect of Altered pH on Macrophage Activity

Cell culture conditions were changed to a CO_2_-free incubator and culture medium pH increased from pH 6.5 to pH 8.5 in 1.0 increments using HCl and NaOH titration. After 48 hour exposure to altered pH conditions, THP-1 macrophages were labeled in 100 μL/million cells in eBioscience™ Flow Cytometry Staining Buffer (Thermo Fisher) with 0.1 μL Zombie Aqua™ (BioLegend, San Diego, CA), 2 μL Brilliant Violet 421™ anti-CD-80 antibody (BioLegend, Cat# 305222), 1 μL APC anti-CD-206 (MMR) antibody (BioLegend Cat# 321110), and 5 μL FITC anti-CD-14 antibody (Abcam Cat# ab28061) prior to fixation and staining using commercial staining and fixation buffers (Thermo Fisher). Positive controls were created by culturing differentiated THP-1 macrophages in 20 ng/mL interferon gamma (IFN-γ) and 1 ng/mL lipopolysaccharide (LPS) for anti-CD-80 and anti-CD-14 and in 20 ng/mL interleukin-4 (IL-4) and 20 ng/mL interleukin-13 (IL-13) for anti-CD-206 in a 5% CO_2_ incubator at pH 7.4 for 48 hours. Samples were analyzed using a FACSAria Fusion (BD Biosciences, San Jose, CA). 10,000 single-cell events were acquired for samples from each treatment group. Samples were gated for live singlet macrophages that were positive for CD14 and the results analyzed using FlowJo v.10 software (BD, Franklin Lakes, NJ)

### Altered pH Treatment in an *Ex Vivo* Liver Model

#### Tissue Procurement and Assurances

Porcine liver was obtained immediately following excision from a USDA-approved abattoir and divided into 10 × 5 × 3 cm sections to create the geometrical domains required. Tissue collection was deemed exempt from Institutional Animal Care and Use Committee review.

*IRE + Cathodic Electrochemical Reactions (CER) delivery.* An ECM 830 Square Wave Electroporator was employed to deliver IRE pulses using a bipolar needle electrode placed in the center of the liver sample at a depth of 4 cm. During IRE delivery current and voltage readings were collected using a wideband current monitor connected to a Wavesurfer 3024z oscilloscope.

**Fig. 1:**
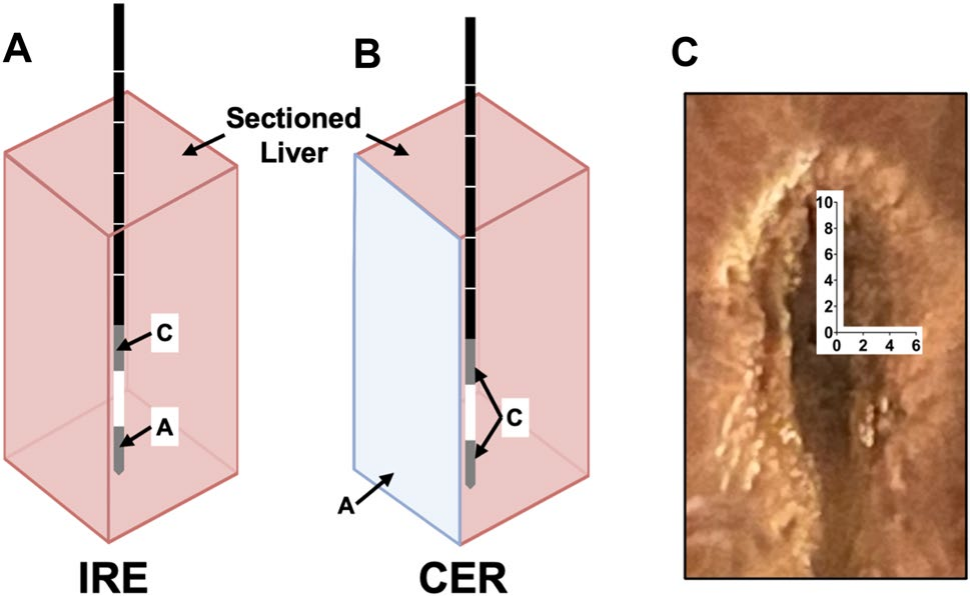
Methods for IRE + CER delivery to liver tissue and subsequent pH measurements. **a** IRE pulse parameters consisted of 100 pulses of 100 μs in length at 1000 V delivered through the cathode **c** and anode **a**, both on the needle electrode. **b** CER pulse parameters consisted of 400 pulses of 10 ms in length at 250 V delivered through the needle electrode (**C**) and a larger ground electrode (**A**). **c** Spatial resolution of pH measurements collected in 2 mm increments from the center point of the electrode tip immediately following IRE + CER treatment.

For tissue subject to IRE + CER, a bipolar needle electrode was connected so that the anode and cathode were both initially on the needle electrode (Fig. 1a*).* Pulse delivery consisted of 100 pulses of 100 μs in length at 1000 V. Immediately after pulse delivery both electrodes on the needle were connected to the cathode and a ground electrode was introduced as the anode (Fig. 1b*)* for pH treatment (400 pulses, 10 ms in length at 250 V, delivered with a measured current of 2.5 A [taken from the average of first and last pulse])*.*

#### pH Measurement in Liver

Immediately following pulse delivery, tissue was sectioned longitudinal to the path of electrode insertion and sequential pH measurements were collected in 2 mm increments from the center point of the electrode tip (Fig. 1c*).* To ensure rapid data collection, measurements were considered symmetrical along each axis and both electrodes were considered as replicates of each other. The pH was also measured at a single point 8 mm radially from the center of the ablation every 15 minutes until the pH reached 6.4 (equal to untreated region). pH measurements were made using an Orion™ 8103BNUWP ROSS Ultra™ pH Electrode (ThermoFisher Scientific).

#### Statistical Analysis

An ordinary one-way ANOVA and post hoc Tukey’s multi-comparison test was used to analyze differences in macrophage viability and activation and effect of pulse length on pH. Pearson’s correlation coefficient was used to analyze effect of increased current and voltage on pH. A *p*-value < 0.05 was considered significant.

## Results

### Effect of Pulse Parameters on pH in Agarose Phantoms

**Fig. 2:**
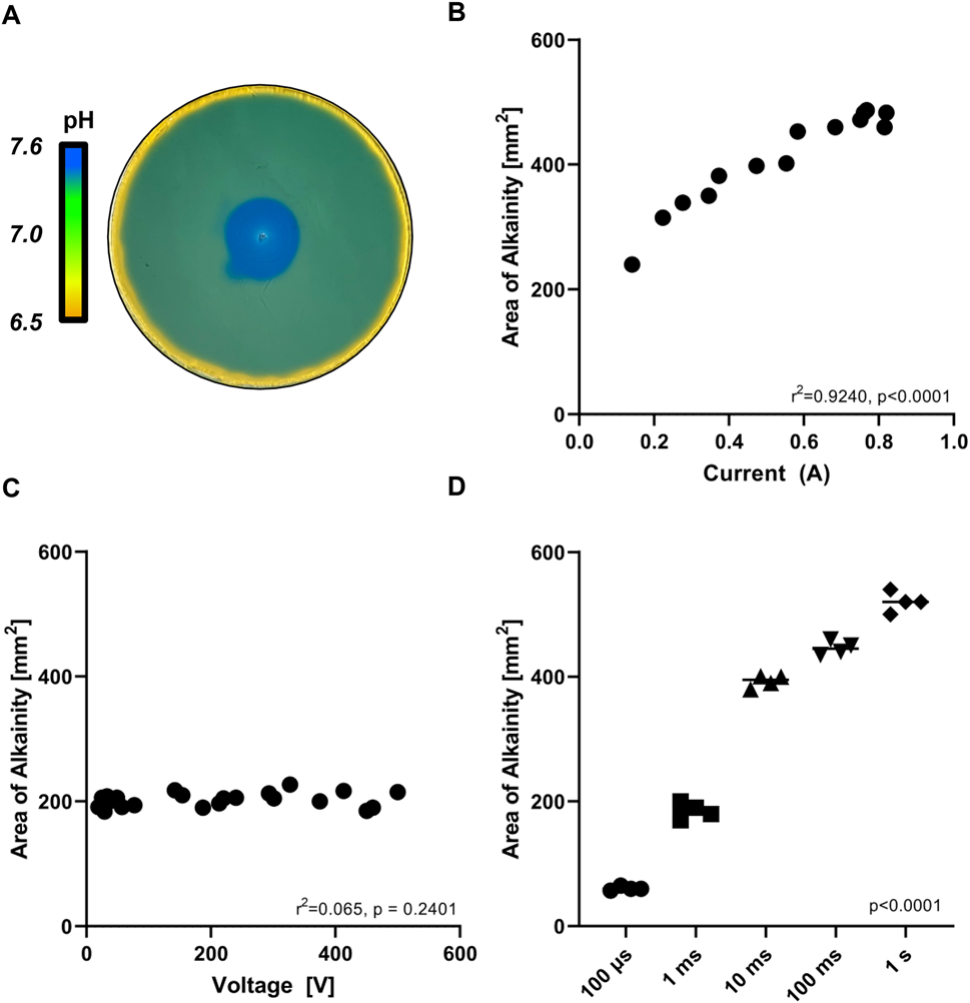
The effect of IRE on pH is regulated by current and pulse width but not voltage. **a** Representative image of agarose phantoms containing bromothymol blue (indicator) following IRE delivery. The change in pH change was defined as the area of the blue circle. **b** Effect of increasing current with constant voltage and pulse length on area of pH change in agarose phantoms containing bromothymol blue (r^2^ = 0.9240, N = 23, *p* < 0.0001). **c** Effect of increasing voltage with constant current and pulse length on area of pH change in agarose phantoms containing bromothymol blue (r^2^ = 0.065, N = 23, *p* = 0.240). **d** Effect of increasing pulse length with constant voltage and current on area of pH change in agarose phantoms containing bromothymol blue (*p* < 0.0001 between all groups)

Application of IRE pulse delivery led to rapid, reproducible changes in pH detected using bromothymol blue (yellow (acidic pH), green (neutral pH), blue (basic pH)) (Fig. 2a). Increasing the current in the setting of a constant voltage led to an increased area of pH change with a maximum pH change at a current of 0.85 A (Fig. 2b, r^2^ = 0.9240, *N* = 14, *p* < 0.0001). Conversely, stepwise increases in voltage in the setting of constant current failed to alter the area of pH change (Fig. 2c, r^2^ = 0.065, *N* = 23, *p* = 0.2401). Changes in pulse length at a constant voltage and current led to sequential increases in area of pH change with a maximal change being detected at a 1 s pulse length (Fig. 2d, p < 0.0001 between all groups).

### Effect of Electrode Geometry on pH Change in Agarose Phantoms

**Fig. 3:**
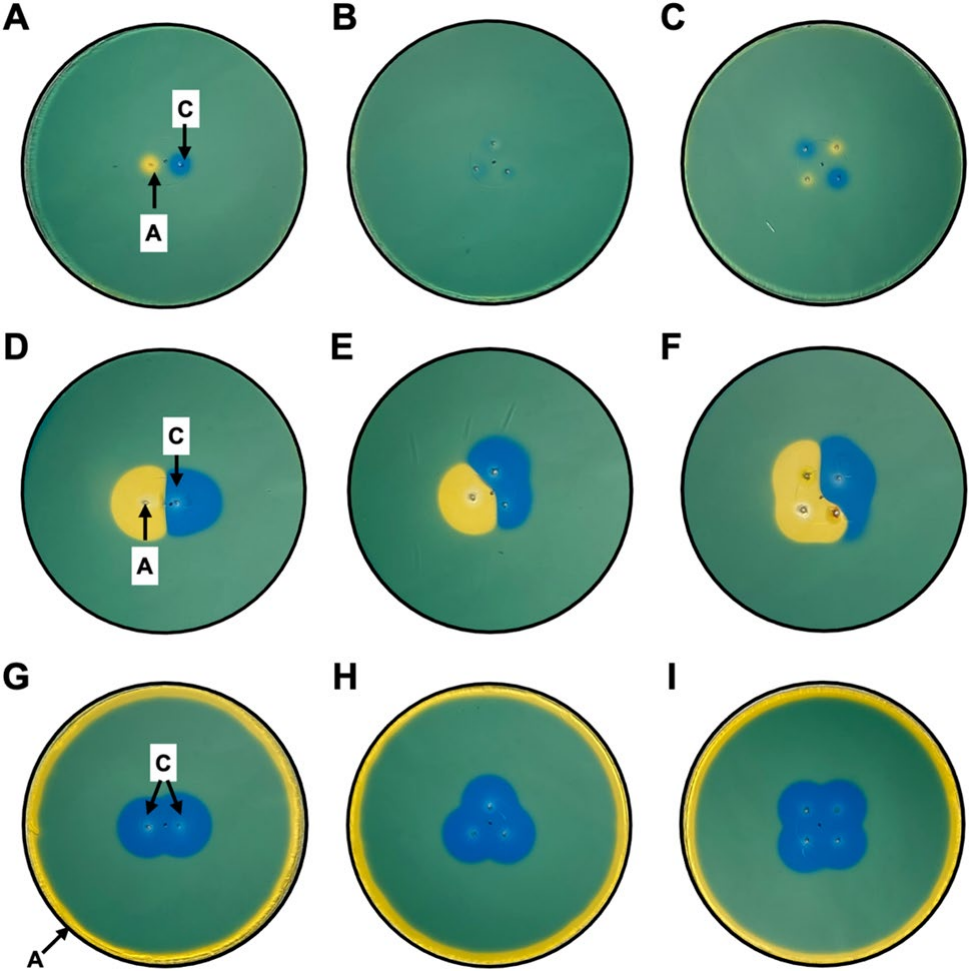
The effect of IRE on pH is regulated by electrode number and geometry. Representative image when **a** Using a two-electrode approach and IRE pulse settings of 100 μs at 1000 V demonstrate the anode (**A**) and cathode (**C**) create small acidic and alkaline regions adjacent to the electrodes. Using **b** 3 and **c** 4 electrode geometries and IRE pulse settings of 100 μs at 1000 V results in ambiguous zones of acidity and alkalinity at the anode and cathode. Representative images using energy matched, lower voltage pulses of longer wavelength (1 s at 10 V) demonstrate the creation of more intense pH effects at the anodes/cathodes when using **d** 2 electrode, **e** 3 electrode, and **f** 4 electrode arrangements. Representative images demonstrate defined regions of alkalinity are achieved when all of the electrodes in the target zone are connected to the cathode and a larger ground electrode serves as the anode when using **g** 2 electrode, **h** 3 electrode, and **i** 4 electrode arrangements.

Using a dual electrode arrangement (100 μs pulse length, 1000 V) resulted in distinct, localized pH changes with the pH adjacent to the anode becoming more acidic (yellow) and the pH around the cathode becoming more basic (blue) (Fig. 3a). Using a 3-electrode arrangement and cycling the cathode-anode arrangement (100 μs pulse length, 1000 V) resulted in less clear changes in pH compared to the 2 electrode arrangement (Fig. 3b), whereas the 4 electrode arrangement resulted in 2 regions of increased pH and 2 regions of decreased pH, albeit with smaller regions of pH change compared to the 2 electrode arrangement (Fig. 3c). Altering the pulse delivery parameter to increase pulse length (1 s) at a lower voltage (10 V) led to an increased area of pH change for all electrode arrangements employed (Fig. 3d–f). When changing the design to incorporate a grounding electrode to serve as the anode, pulse delivery (1 s, 10 V) led to sustained pH increases around the electrodes for all arrangements employed (Fig. 3g–i).

### Effect of pH on Macrophage Viability and Activation

**Fig. 4:**
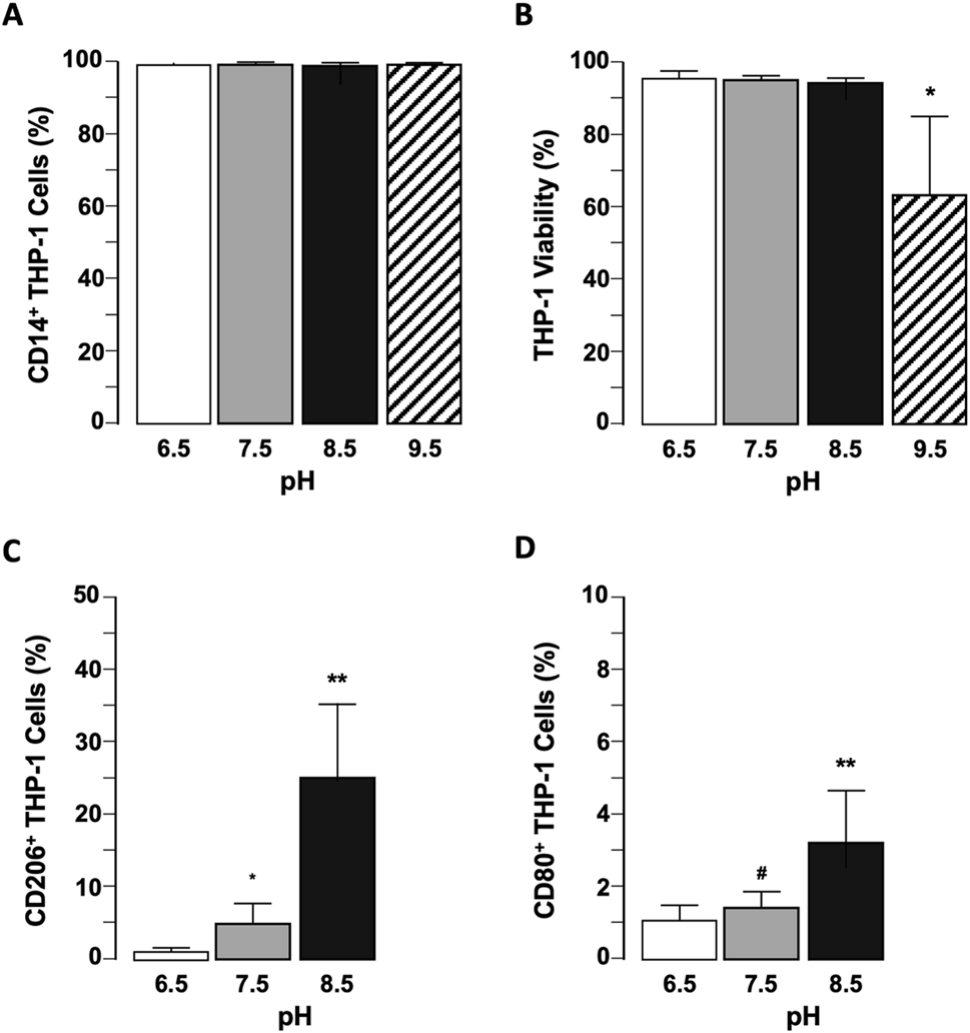
Viability and activation of THP-1 macrophages are affected by culture medium pH. **a** Effect of increasing pH on THP-1 macrophage CD14 expression detected in viable THP-1 cells using flow cytometry analysis. *p* = 0.1678 (ns). **b** Effect of increasing pH on THP-1 macrophage cell viability. *p < 0.01 versus all other pH levels tested, *n* = 6 independent experiments. **c** Effect of increasing pH on THP-1 macrophage CD206 expression (M2 activation marker) detected in viable THP-1 cells using flow cytometry analysis. **p < 0.0001 versus pH 7.5 and pH 6.5, *p < 0.01 versus pH 6.5, *n* = 6 independent experiments. **d** Effect of increasing pH on THP-1 macrophage CD80 expression (M1 activation marker) detected in viable THP-1 cells using flow cytometry analysis. *p < 0.0001 versus pH 7.5 and pH 6.5, ^#^*p* = 0.9094 (ns) versus pH 6.5, *n* = 6 independent experiments.

After gating for live-dead cells, macrophage (THP-1) cell population purity was confirmed (CD14 staining) (Fig. 4a). Increasing culture medium to pH > 8.5 led to decreased THP-1 cell viability (Fig. 4b, n = 6 independent experiments, *p* < 0.01 pH 9.5 *versus* all other pH levels tested) and an increased percentage of THP-1 cells staining positive for CD206 (tumor-associated macrophage marker) (Fig. 4c, n = 6 independent experiments, *p* < 0.0001 pH 8.5 *versus* pH 7.5 and pH 6.5) and CD80 (marker of M1 activation) (Fig. 4d, n = 6 independent experiments, *p* < 0.0001 pH 8.5 *versus* pH 7.5 and pH 6.5), albeit with a higher percentage of cells staining for CD206 than CD80 at pH 8.5 (Fig. 4c and d).

### Effect of IRE on Tissue pH in *Ex Vivo* Liver Tissue

**Fig. 5:**
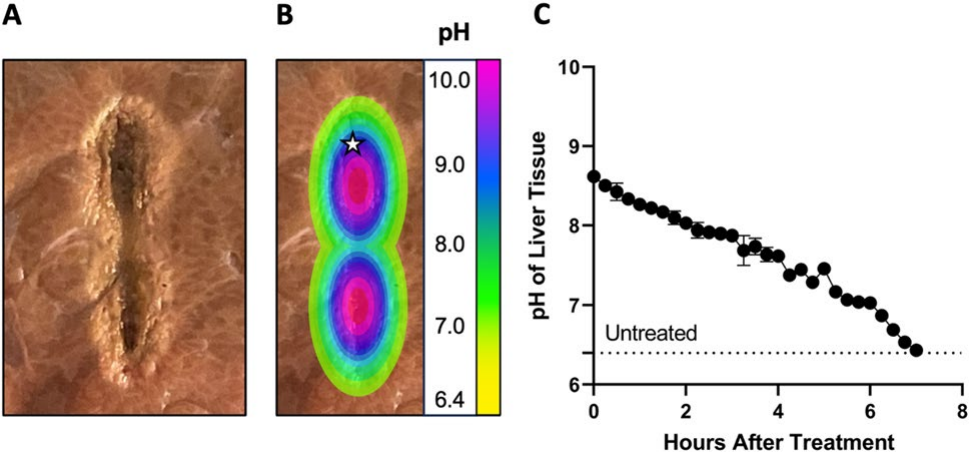
Tissue pH can be increased using IRE pulses *ex vivo*. **a** Representative image depicting a tissue (liver) slice following IRE delivery highlighting a necrotic (darker) center (darker) and outer transition zone. **b** Representation of changes in pH zones measured experimentally and superimposed across the tissue slice **c** Change in pH in tissue over time following IRE delivery measured at highlighted point (star) in figure B. *n* = 3 independent experiments.

Using a single needle, dual electrode bipolar device ablations were readily detectable in *ex vivo* porcine liver tissue following pulse delivery (100 pulses, 100 μs length, 1000 V + 400 pulses, 10 ms length, 250 V) (Fig. 5a). Measurement of tissue pH immediately following IRE + CER delivery using tissue sectioning demonstrated pH remained significantly elevated up to 12 mm from the center of each needle electrode in the x- and y-planes (Fig. 5b and Table 1). Periodic measurement of pH at 15 min intervals at the margin of basic-neutral tissue reveal pH remained significantly elevated compared to normal tissue up to 7 hours after initial IRE + CER delivery (Fig. 5c*)*

**Table 1:** pH of porcine liver treated with IRE + CER

y-axis distance (mm)	pH	x-axis distance (mm)	pH
0	10.5 ± 0.3	0	10.5 ± 0.3
2	10.2 ± 0.2	2	9.9 ± 0.3
4	9.5 ± 0.4	4	8.9 ± 0.1
6	9.2 ± 0.3	6	8.7 ± 0.2
8	8.9 ± 0.4	8	8.3 ± 0.1
10	8.6 ± 0.4	10	7.2 ± 0.3
12	8.3 ± 0.1	12	6.8 ± 0.5
14	7.5 ± 0.3	14	6.4 ± 0.0
16	7.0 ± 0.5	16	6.4 ± 0.0
18	6.4 ± 0.0	18	6.4 ± 0.0

## Discussion

The pH of the TME differs from that of nontumor tissue due to altered tumor metabolic activity, and changes in TME pH can function to alter tumoral immune responses. Electroporation-based ablation therapies (IRE) are growing in popularity and initiate a robust immune response following treatment.^44^ To date, the electrochemical effects arising from IRE have largely been considered incidental (outside of electrolytic electroporation). Data presented herein demonstrates it is possible to control pH to a physiologically relevant magnitude in a sizeable area for a sustained amount of time by modulating IRE pulse delivery approaches in model systems.

Using agar phantoms with differing NaCl content to produce constant current demonstrated, that even as voltage increased by as much as 50-fold, pH remained unchanged. Conversely, when voltage was maintained and current altered in the same experimental system, a correlation of current to pH was detected in a manner similar to that previously reported,^21^ as well as a correlation of pulse width to pH. Current and pulse width can be described by the charge equation, where Q is charge, I is current, $\tau$ is pulse length, and N is pulse number. Charge dictates the total amount of electrons delivered or removed from the system, and these electrons drive the electrochemical reactions at the electrode and tissue interface. At the cathode, water is reduced to hydrogen gas and produces hydroxide ions, ultimately increasing the tissue’s pH. Conversely, at the anode water is oxidized to oxygen gas and produces hydrogen ions, decreasing the local pH. Our data, in agreement with existing literature, indicates the pH change during IRE delivery is proportional to the total charge delivered.

$$\Delta pH\propto Q=I* \tau *N$$

From a practical perspective, when using IRE clinically these data are relevant since the two most commonly altered pulse delivery parameters are voltage and pulse width. These data from a model system suggest that to successfully manipulate tissue pH within the ablation focus should be directed toward pulsing paradigms designed to deliver lower voltage pulses of longer duration. Additionally, previous studies report that these types of pulse deliveries have the added advantage of the diminished potential for nerve excitation and thermal damage.^5^ Similar to the pH change, neuromuscular stimulation is weakly dependent on current and pulse width. However, an important distinction is that the neuromuscular stimulation is primarily dependent on the pulse amplitude, whereas the pH change is dependent on the current amplitude, pulse width, and the number of pulses. Therefore, we speculate the use of high frequency (i.e., very short duration) monophasic pulses, delivered in a high number over time, could combat neuromuscular stimulation without sacrificing pH change.

An important consideration when performing *in situ* tissue ablation is the ability to accurately predict the ablation zone *in vivo*. Previous studies using commercial IRE systems report the ablation size and shape depend on both the pulse parameters delivered and the number/arrangement of the electrodes used. In the simplest case this involves placing electrodes in parallel on either side of the target lesion. However, by increasing the number of electrodes used, the possibility arises to alter both the positioning in and/or around the target and the sequence in which the pulses are delivered between the respective electrodes. When modeling this approach using agar phantoms we demonstrated that when using 2 parallel electrodes a balance of acidic and alkaline pH changes occurs between the anode and cathode. To address this imbalance in pH change and create a uniform pH change we connect multiple electrodes to the cathode terminal within the target region in conjunction with a separate anode of much greater surface area relative to the cathodes. This allows for sustainable changes in pH in the targeted region in conjunction with minimal changes in pH at the larger anode. Such an effect is explainable using Kirchhoff’s circuit laws which states the current entering the domain must equal the current exiting the region. Thus, it should be possible to manipulate both the size and shape of the pH change region by altering the number or the geometry of the electrodes within the target region when using a distant surface electrode.

In considering such an approach *in vivo*, the effect of DC pulses on neuromuscular activity should be closely monitored as electrical current will flow through skeletal muscle and neurons to the grounding pad raising the possibility of muscle twitch/spasm. In the simplest translation to the clinic, this treatment requires minimal additional work from the surgical team. A bipolar needle electrode can be left *in situ* following IRE, and a surface electrode, connected to the anode, will be fastened to the patient before CER treatment is delivered through the needle electrode. Importantly, the tissue in proximity to the anode will be exposed to acidic reactions. While we expect this change to be minimal in comparison to the pH change at cathode, the use of inert electrode materials and electrode coatings can further mitigate acidosis.^12^ Also, the surface in contact with the grounding pad could be primed with an alkaline gel to offset the acidosis. Along with these methods to mitigate incidental pH changes, the data in Fig. 4b suggests we will not have significant loss in viability within this range. However, the data are limited to macrophages, so further investigations are needed for the effect of a pH change on liver cells and tissue.

In developing approaches to alter the TME pH to activate anti-tumor immune responses, there is an inherent need to identify an optimal target pH to do so. In this study we employed an *in vitro* macrophage (THP-1 cells) model by activating M0 (uncommitted) monocytes using PMA (which differentiates the cells into macrophages). However, these cells remain plastic and can maintain polarization (M1) or re-polarize (M2) based on changes in the milieu of inflammatory mediators and cell culture conditions (i.e., IFN- γ and LPS for M1; IL-4 and IL-13 for M2). Once the M1/M2 state was confirmed (FACS), a pH of ≥ 8.5 was more effective than lower pH (neutral or acidic) in polarizing M0 macrophages. Specifically, we identify that CD206 expression increases as the alkalinity of culture medium rises. However, while CD206 is usually considered a tumor-associated macrophage (TAM) marker, it can also indicate an anti-tumor response.^15,23^ This was confirmed in our model system by a parallel increase in CD80 expression as pH increased.^24,40^ While care should be taken in interpreting these *in vitro* data relative to the more complex *in vivo* environment, it is tempting to speculate that the changes in polarity induced by elevating pH could impact resident TAMs and monocytes entering the TME by extravasation. Equally, detailed *in vitro* and *in vivo* studies are required to fully evaluate the effect of changes in pH on M1 *versus* M2 transition in macrophages.^10^

Unlike thermal ablation, in which *ex vivo* tissue ablations closely mimic those achieved *in vivo*,^27^ the dynamic cell death processes induced following IRE delivery can only be fully modeled *in vivo*. However, perfused and nonperfused tissues have been employed to demonstrate “proof of concept” to model *in vivo* efficacy.^29,30^ As previously reported use of a dual electrode – single needle IRE delivery device results in a peanut shaped ablation due to the bipolar electrode geometry in which the effects of IRE are more pronounced at the electrodes compared to the insulation between them ^17^. The center, darker region immediately adjacent to the electrodes typically undergoes necrotic, thermal damage.^38,39,45^ Based on these previous studies, the extent of the IRE ablation should extend to the paler region, with the region beyond this experiencing reversible electroporation. Based on our data, the pH treatment created a region of alkalinity along the transition zone between irreversible and reversible electroporation, which may prove beneficial if the anti-tumor immune response is enhanced by increasing pH as this region is the most likely site of incomplete ablation and local recurrence.^28^ Of further note, the initial pH of the excised liver in our study was 6.4, a pH that is similar to that reported in the TME, and the pH of the transition region took 7 hours post IRE + CER delivery to return to pH of 6.4. However, as previously highlighted, the maintenance of this pH change may be exaggerated in the absence of tissue perfusion and detailed *in vivo* analysis is required to determine the relevance to *in vivo* IRE ablation.

In considering and interpreting the data presented, several important limitations should be considered. Firstly, while the use of agar phantoms and *ex vivo* tissue provide critical preclinical data regarding the potential to manipulate the TME pH, this should be balanced against the anatomical and pathophysiological complexities associated with the TME *in vivo*. Although the *ex vivo* liver tissue provides a suitable domain for this initial study of the pH gradient, the use of a perfused liver model in a future study would provide insight to the spatial and temporal changes seen *in vivo*. Similarly, analyzing the effect of pH on macrophage phenotype provides promising preliminary data regarding the potential of inducing anti-tumor immune responses in the TME. However, we stress that many tumors (including hepatomas) arise in the setting of damaged or compromised non-tumor tissue in which the immune cell component can (and often is) profoundly different to that which exists in healthy tissue.^6,26,43^ Finally, it is important to reiterate that, following IRE delivery, the ablation zone is a dynamic physiological environment in which numerous immunological and non-immunological pathways (including programmed cell death) interact. Thus, to fully understand the impact of changing tissue pH using IRE on ablation efficacy will require detailed *in silico, ex vivo* and *in vivo* experimental approaches.

A major limitation associated with cancer therapy is the TME which is overall immunosuppressive, in part due to its acidity. The preclinical data presented herein indicate that changing IRE ablation parameters can be utilized to increase regional pH changes within the post-IRE ablation zone. Given the role of pH on determining macrophage phenotype and the impact of macrophages and the immune response in tumor progression, these data suggest that modulating TME pH through electrolysis could function to complement IRE treatment by enhancing the anti-tumor immune response.
