# Correction: Harnessing the Electrochemical Effects of Electroporation-Based Therapies to Enhance Anti-tumor Immune Responses

**DOI:** 10.1007/s10439-024-03444-w

**Published:** 2024-01-10

**Authors:** Zaid S. Salameh, Kenneth N. Aycock, Nastaran Alinezhadbalalami, Khan Mohammad Imran, Iain H. McKillop, Irving C. Allen, Rafael V. Davalos

**Affiliations:** 1 Department of Biomedical Engineering and Mechanics, Virginia Tech, 325 Stanger St, Blacksburg, VA 24061 USA; 2 Department of Biomedical Sciences and Pathobiology, VA-MD College of Veterinary Medicine, Virginia Tech, 205 Duck Pond Drive, Blacksburg, VA 24061 USA; 3 Department of Surgery, Atrium Health Wake Forest Baptist Medical Center, 1000 Blythe Blvd, Charlotte, NC 28203 USA; 4 Wallace H. Coulter Department of Biomedical Engineering, Georgia Tech - Emory, 313 Ferst Dr, Atlanta, GA 30308 USA

**Correction to: Annals of Biomedical Engineering (2023) 52:48–56** 10.1007/s10439-023-03403-x

The article Harnessing the Electrochemical Effects of Electroporation‑Based Therapies to Enhance Anti‑tumor Immune Responses, written by Zaid S. Salameh, Kenneth N. Aycock, Nastaran Alinezhadbalalami, Khan Mohammad Imran, Iain H. McKillop, Irving C. Allen, and Rafael V. Davalos, was originally published electronically on the publisher’s internet portal on November 21, 2023 without open access. With the author(s)’ decision to opt for Open Choice the copyright of the article changed on December 21, 2023 to © The Authors 2023 and the article is forthwith distributed under a Creative Commons Attribution CC BY.

